# Digitally Guided Direct Composite Rehabilitation of a Maxillary Central Incisor Crown Fracture Associated With Multiple Diastemas: A 24‐Month Clinical Case Report

**DOI:** 10.1002/ccr3.73783

**Published:** 2026-10-22

**Authors:** Chang Liu, Binghua Lv, Huimin Nie, Xiangyu Ji, Zhishan Lu

**Affiliations:** ^1^ School of Stomatology Shandong Medical and Pharmaceutical University Yantai Shandong People's Republic of China; ^2^ Department of Endodontics Stomatological Hospital of Shandong Medical and Pharmaceutical University Yantai Shandong People's Republic of China; ^3^ Yantai Engineering Research Center for Digital Technology of Stomatology Yantai Shandong People's Republic of China; ^4^ Characteristic Laboratories of Colleges and Universities in Shandong Province for Digital Stomatology Yantai Shandong People's Republic of China

**Keywords:** anterior diastema, crown fracture, digital dentistry, direct composite resin, minimally invasive dentistry, target restoration space

## Abstract

Digitally guided TRS‐based direct composite layering allows predictable, minimally invasive rehabilitation of crown fractures associated with multiple diastemas, offering a conservative alternative when orthodontic or indirect restorative treatments are not feasible.

## 1 Introduction

Anterior tooth defects and interdental spacing are common esthetic challenges in restorative dentistry, with significant impact on smile harmony, facial esthetics, and patient psychosocial well‐being [1, 2]. Traumatic crown fractures further aggravate these problems by causing loss of tooth structure and disruption of anterior dental harmony. When combined with anterior spacing, restoration becomes more complex, particularly in patients seeking conservative treatment.

Several treatment options are available, including orthodontic therapy, direct composite resin restorations, and indirect ceramic restorations. While these approaches have been demonstrated to restore both function and esthetics, they differ in terms of treatment duration, invasiveness, cost, and biological impact. Indirect restorations provide predictable esthetic outcomes but require greater tooth preparation [3, 4, 5], while orthodontic treatment allows space redistribution but cannot restore lost tooth structure and requires prolonged treatment time. Therefore, treatment selection should be individualized based on clinical conditions and patient expectations.

Direct composite resin restoration is widely used as a minimally invasive and reversible approach for anterior rehabilitation [6]. However, in complex cases involving crown fractures and multiple diastemas, freehand techniques may fail to achieve precise space redistribution and harmonious proportions. This can potentially result in overcontoured restorations, unfavorable emergence profiles, and black triangles, thereby compromising esthetic predictability.

Recent advancements in digital dentistry, including intraoral scanning, facial analysis, and three‐dimensional virtual planning, have enhanced preoperative visualization and restorative accuracy [7]. In addition, digital wax‐ups and silicone index transfer techniques enhance clinical execution and improve treatment predictability [8]. The integration of digital space analysis with direct composite layering may therefore support more precise management of complex anterior esthetic cases while maintaining a minimally invasive approach.

However, clinical evidence regarding the application of digitally guided space analysis combined with direct composite restoration for crown fractures associated with pre‐existing anterior spacing remains limited. This case report describes a digitally assisted direct composite approach for the functional and esthetic rehabilitation of a maxillary central incisor fracture associated with multiple diastemas. The approach focuses on tooth morphology, color, and proximal relationships.

## 2 Case History/Examination

A 34‐year‐old female patient presented with esthetic concerns following traumatic fractures of the right and left maxillary central incisors. The patient reported that the pre‐existing anterior spacing had become more noticeable after the trauma, leading to dissatisfaction with the overall appearance of her anterior dentition. Clinical examination revealed an oblique crown fracture of the right maxillary central incisor extending to the middle third of dentin, partial enamel loss at the incisal edge of the left maxillary central incisor, multiple anterior spacing, and disharmonious anterior tooth proportions (Figure 1A–E). The patient requested minimally invasive esthetic rehabilitation and reported no relevant medical history or drug allergies. The affected tooth was asymptomatic, with no tenderness to percussion, no mobility, and normal response to pulp vitality testing. Radiographic examination confirmed the absence of root fracture, periapical radiolucency, or significant alveolar bone loss (Figure 1F). A comprehensive evaluation, including intraoral examination, clinical photography, radiographic assessment, and digital scanning, was performed.

**FIGURE 1:**
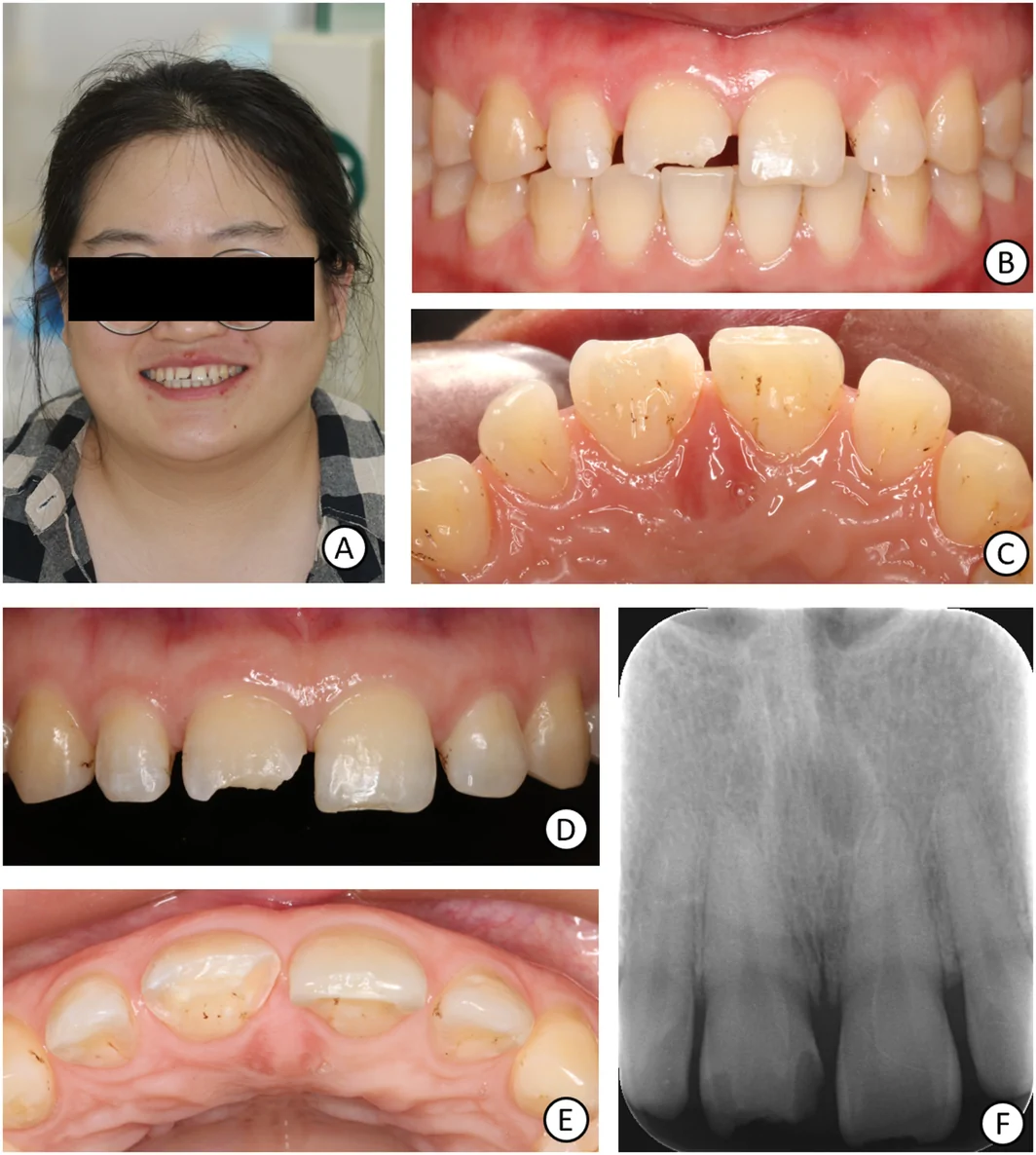
Initial situation. (A) Facial examination, (B–E) Oral examination, (F) Imaging examinations.

## 3 Differential Diagnosis, Investigation and Treatment

The patient was diagnosed with traumatic crown fractures of the bilateral central incisors, associated with multiple anterior diastemas in the maxillary dentition. The patient was informed of all available treatment options, including direct composite resin layering, porcelain veneer restoration, orthodontic treatment combined with restorative therapy, and full‐coverage crown restoration. In consideration of the patient's age, the minimal extent of tooth structure loss, the advantageous pulpal and periapical conditions, and their predilection for a minimally invasive, expeditious esthetic solution, a digitally assisted direct composite resin layering approach was selected through a collaborative decision‐making process. Digital restorative planning, in conjunction with restorative space analysis, was employed to ascertain the requisite restorative volume for each anterior tooth. The treatment plan was subsequently implemented in a clinical setting, employing a diagnostic wax‐up and a silicone index as essential tools. Prior to the restorative treatment, the patient underwent basic periodontal preparation to optimize the gingival environment.

A digital workflow was implemented for preoperative analysis, integrating intraoral scanning (TRIOS Intraoral Scanner, 3Shape A/S, Copenhagen, Denmark) (Figure 2A,B), facial scanning (MetiSmile 3D Facial Scanner, SHINING 3D Technology Co. Ltd., Hangzhou, China), and CBCT data. Three‐dimensional intraoral data of the maxillary and mandibular arches and occlusion were obtained using the intraoral scanner, while facial soft tissue data were acquired using the facial scanner. The datasets were exported in DCM and OBJ formats, respectively. All data were imported into dental CAD software (exocad Dental CAD Software, exocad GmbH, Darmstadt, Germany) for registration to construct a three‐dimensional virtual patient model (Figure 2C,D). Subsequently, a digital diagnostic wax‐up was conducted (Figure 2E,F).

**FIGURE 2:**
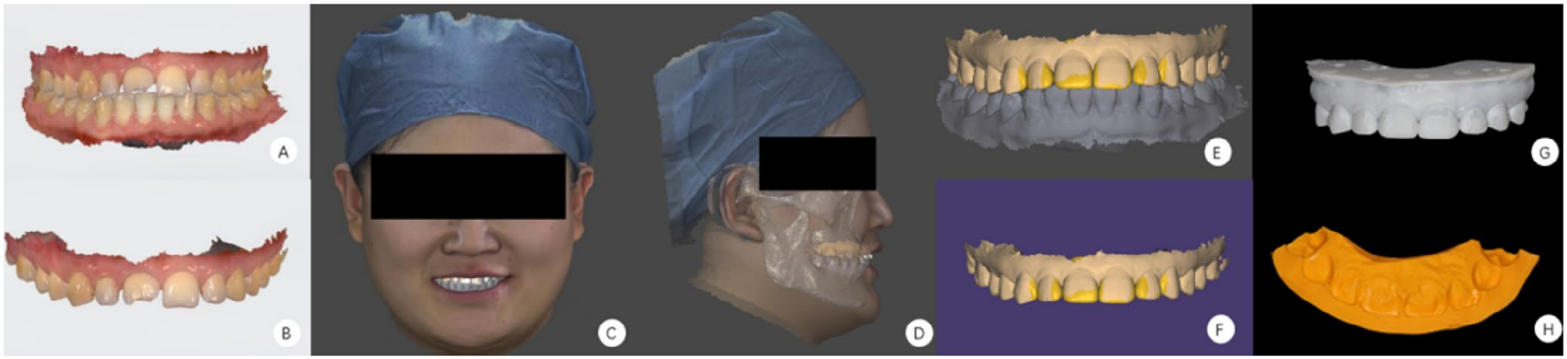
The virtual patient and digital esthetic restorative design. (A, B) Intra‐oral scan, (C, D) 3D virtual patient, (E, F) digitally designed maxillary restorations, (G) 3D printed diagnostic esthetic wax‐up, (H) TRS‐guided silicone index.

Within the virtual model, the following parameters were evaluated: tooth morphology, interdental space distribution, anterior tooth proportions, midline position, and occlusion in the canine‐to‐canine region. The target restoration space (TRS) values were calculated for each anterior tooth (Figure 3) based on the planned restorative contours to guide restorative planning. A digital diagnostic wax‐up was fabricated according to the virtual design and produced using a three‐dimensional printer (UltraCraft ChairSide Pro 3D Printer, HeyGears Technology Co. Ltd., Guangzhou, Guangdong, China) (Figure 2G). A silicone index was subsequently fabricated using a silicone impression material (Silagum Silicone Impression Material, DMG Chemisch‐Pharmazeutische Fabrik GmbH, Hamburg, Germany) to reproduce the palatal and incisal morphology of the maxillary canine‐to‐canine region, thereby creating a TRS‐guided silicone index (Figure 2H). The index was employed during the chairside procedure to transfer the virtually planned palatal contours, incisal edge position, and proximal boundaries. This enhanced the controllability of the direct composite resin layering procedure.

**FIGURE 3:**
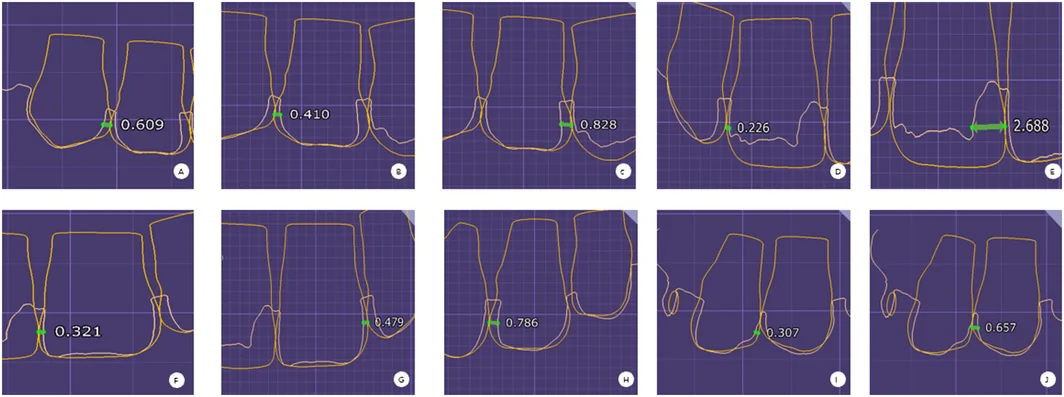
Target restoration space determined by digital esthetic analysis. (A) Mesial TRS of tooth 13, (B) Distal TRS of tooth 12, (C) Mesial TRS of tooth 12, (D) Distal TRS of tooth 11, (E) Mesial TRS of tooth 11, (F) Mesial TRS of tooth 21, (G) Distal TRS of tooth 21, (H) Mesial TRS of tooth 22, (I) Distal TRS of tooth 22, (J) Distal TRS of tooth 22.

Subsequent to the implementation of rubber dam isolation, minimally invasive tooth preparation was conducted. Irregular and weakened enamel margins were meticulously removed while preserving the integrity of the sound tooth structure. The etching of the enamel was carried out using 35% phosphoric acid (GLUMA Etch 35 Gel, Kulzer GmbH, Hanau, Germany) for a duration of 30 s. This was followed by rinsing and air‐drying until a frosty white appearance was discernible. Subsequently, a universal adhesive system (3 M Scotchbond Universal Adhesive, 3 M ESPE, St. Paul, MN, USA) was applied and exposed to light for 20 s. A silicone palatal index (Silicone Index, fabricated from diagnostic wax‐up; DMG Chemisch‐Pharmazeutische Fabrik GmbH, Hamburg, Germany) was positioned to guide palatal contour reconstruction. A 0.5 mm enamel layer was applied using enamel shade composite resin UE2 (ENA HRi Composite Resin, Micerium S.p.A., Avigliana, Italy) and subsequently light‐cured. Subsequent to the removal of the silicone index, additional light curing was performed. A proximal matrix system (Kangtzheng Proximal Matrix System, Huanghua Kangtian Medical Devices Co. Ltd., Cangzhou, Hebei, China) was subsequently placed, and interdental spaces were restored using an incremental layering technique with dentin shade UD3 and enamel shade UE2 composite resins (ENA HRi Composite Resin, Micerium S.p.A., Avigliana, Italy). The final contouring was performed using diamond burs (DIA‐BURS Diamond Bur, MANI, INC., Utsunomiya, Tochigi, Japan) to eliminate occlusal interferences. The surface refinement process involved the use of aluminum oxide polishing discs (Super‐Snap X‐Treme Polishing Disk, Shofu Inc., Kyoto, Japan) for the removal of superficial imperfections, while proximal surfaces were meticulously polished using specialized polishing strips. The final high‐gloss polishing was achieved using polishing wheels (EVE Dental Polishing Wheel, EVE Ernst Vetter GmbH, Keltern, Germany), resulting in smooth margins and anatomically favorable proximal contours (Figure 4). The clinical workflow detailing each step of the treatment process is summarized in Figure 5.

**FIGURE 4:**
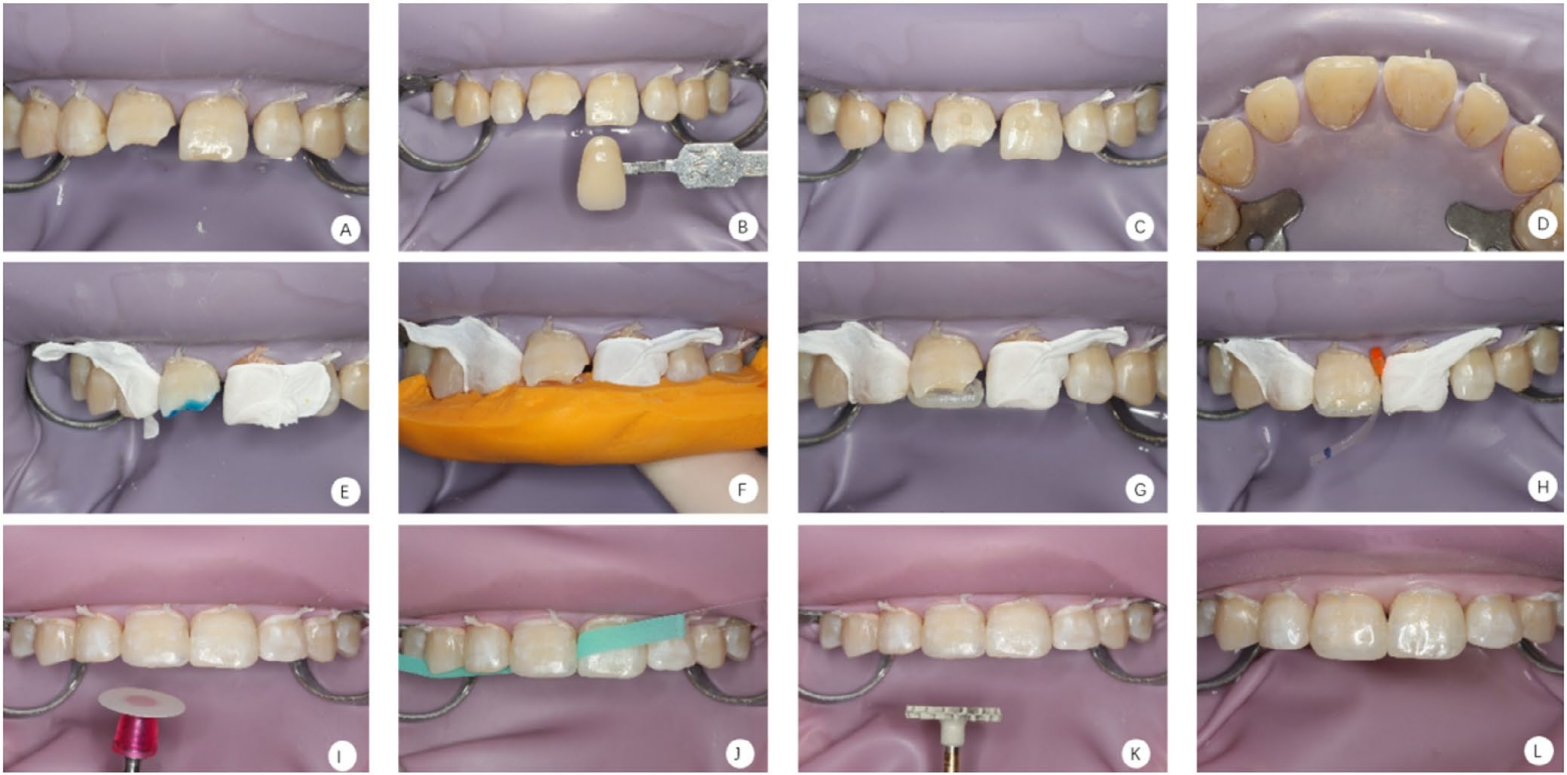
Esthetic restoration using the direct composite resin layering technique. (A) Placement of the rubber dam, (B) Shade matching: Dentin shade 2 M2, (C) Shade matching with composite resin samples, (D) Tooth surface preparation, (E) Etching, (F) Restoration of the palatal surface using a silicone index, (G) Palatal composite resin layering, (H) Restoration of the mesial proximal surface, (I) Sequential polishing, (J) Proximal surface polishing, (K) Polishing using the TWIST polishing system, (L) Facial view after composite resin restoration.

**FIGURE 5:**
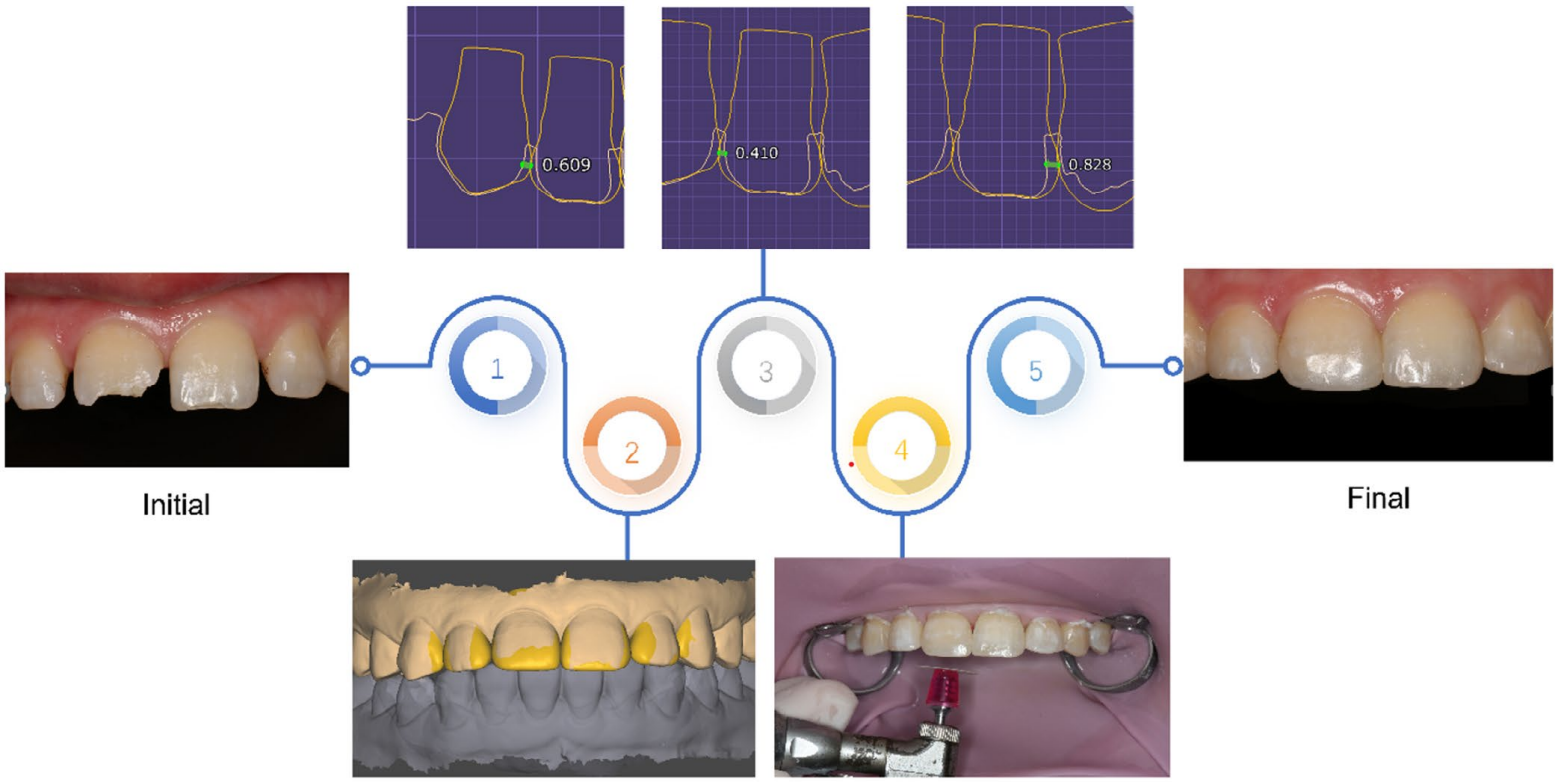
Digital workflow performed in this case study. (1) Initial situation, (2) Digitally designed maxillary restorations, (3) Target restoration space determined by digital esthetic analysis, (4) Esthetic restoration using the direct composite resin layering technique, (5) Final result.

## 4 Results

The advent of digital technology has facilitated the development of a three‐dimensional virtual patient model and preoperative esthetic treatment planning. In the present case, the digitally guided direct composite layering technique was clinically implemented to restore the form, function, and appearance of the anterior dentition (Figure 6A–C).

**FIGURE 6:**
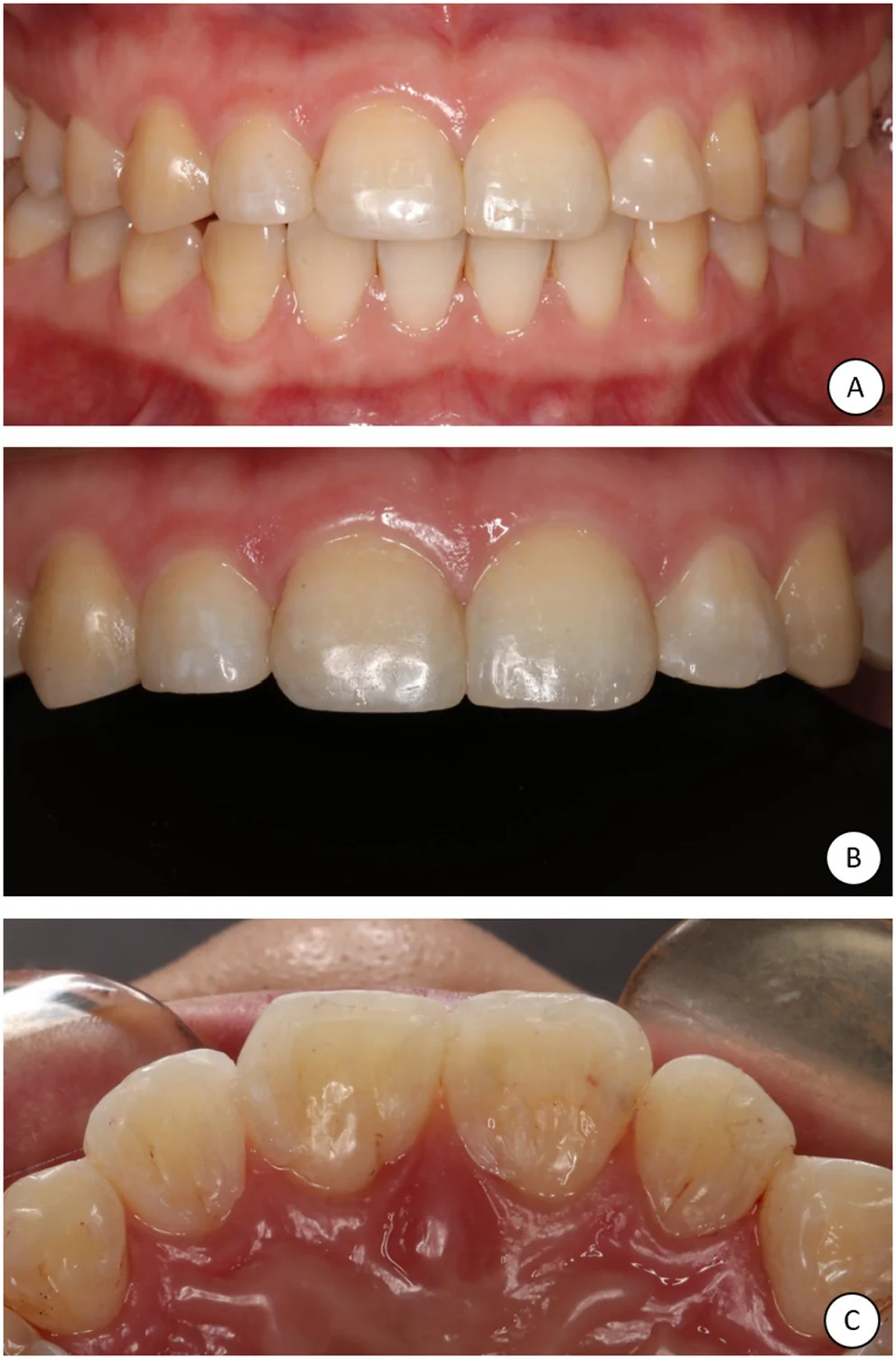
Immediate postoperative intraoral photographs. (A) Postoperative occlusal view. (B) Frontal view of the maxillary anterior teeth. (C) Postoperative palatal view.

At the 24‐month follow‐up, all restorations in the maxillary anterior region remained intact with well‐adapted and continuous margins (Figure 7A–G). No clinically detectable marginal gaps, overhangs, fractures, debonding, or obvious material loss were observed. The restorations maintained their clinical appearance, surface integrity, and anatomical form during the observation period, although no quantitative wear or color analyses were performed. Periodontal examination revealed healthy gingival tissues with normal contours, no signs of inflammation, and no bleeding on probing. No food impaction or occlusal interference during protrusive and lateral movements was detected. The patient reported satisfaction with the esthetic appearance, function, and comfort of the restorations.

**FIGURE 7:**
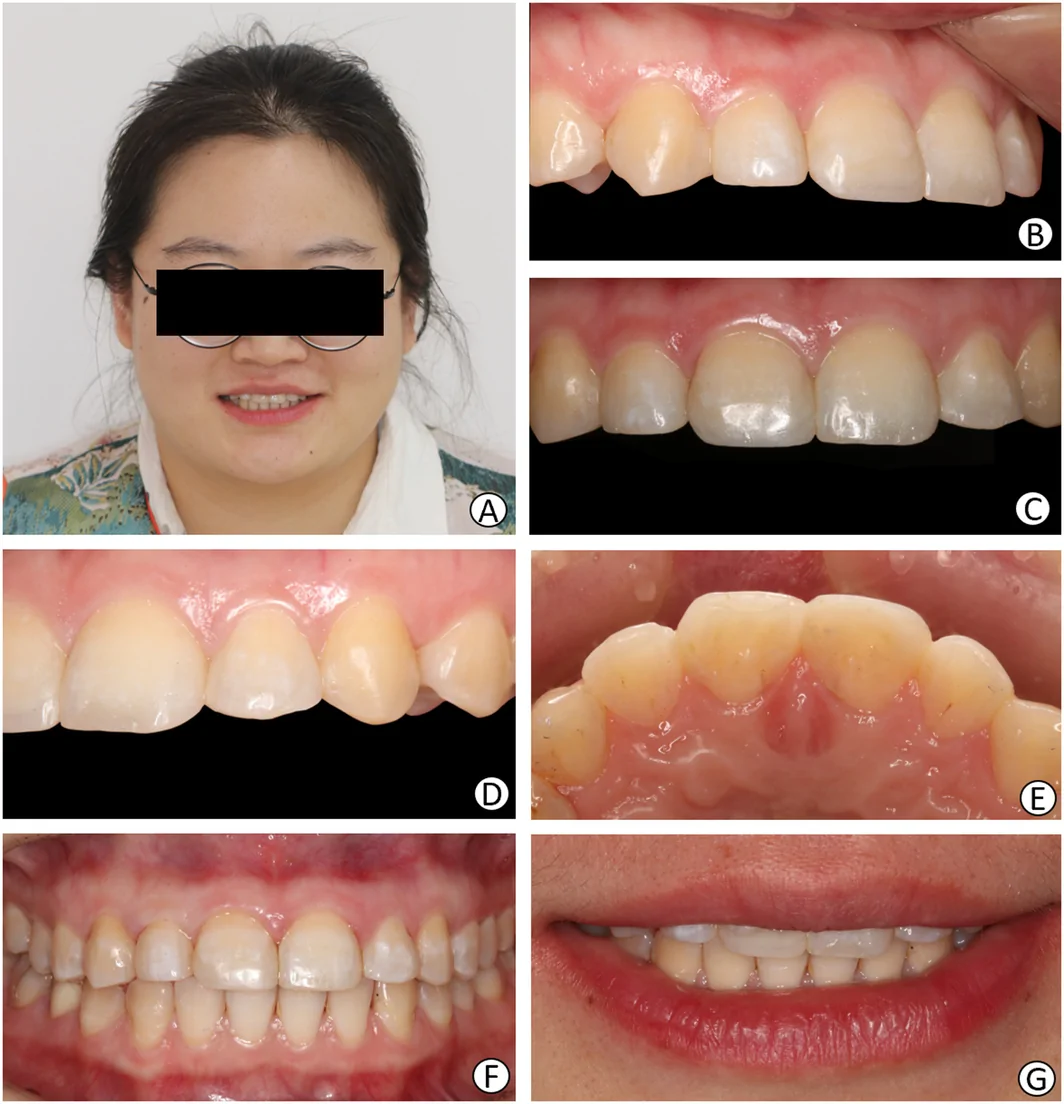
Clinical follow‐up examination at 24 months after treatment. (A) Facial photograph (B–F) Intraoral photographs (G) Lip–tooth relationship photograph.

## 5 Discussion

This case exemplifies a minimally invasive management strategy for a maxillary central incisor crown fracture associated with multiple anterior spacing, employing a digitally guided direct composite approach. The primary clinical challenge entailed the simultaneous restoration of fractured tooth structure and the achievement of controlled redistribution of multiple interdental spaces, while maintaining anterior esthetic harmony. In such complex situations, conventional direct composite techniques are highly operator‐dependent. Intraoperative adjustments have the potential to compromise the predictability of tooth proportions and spatial relationships [9].

A critical component of treatment planning entailed the classification of anterior spacing. After excluding the presence of periodontal disease, occlusal trauma, supernumerary teeth, and other pathological conditions through clinical and radiographic assessment, stable developmental diastemas were diagnosed [10]. This distinction is critical, as only stable spaces can be predictably managed through restorative redistribution without orthodontic intervention.

The treatment planning process was significantly impacted by the patient's decision to decline orthodontic and indirect restorative alternatives. Consequently, a conservative restorative approach was selected to balance esthetic demands, biological preservation, and patient preference. The indication for direct composite resin restoration was based on several factors, including the patient's favorable periodontal health, the presence of sufficient bonding substrates, and the ability to control the total space discrepancy. This allowed for restorative redistribution within both biological and esthetic limits.

The management of multiple diastemas necessitates precise control of spatial distribution, a feat that is often challenging to achieve through freehand techniques alone. In this particular instance, a digital workflow was employed to preoperatively delineate the restorative outcome. The target restoration space (TRS) concept facilitated a structured allocation of restorative volume across the anterior segment, thereby transforming spatial correction from intraoperative estimation into a preplanned digital strategy. This approach enhanced the predictability of tooth proportions and interproximal relationships, thereby reducing operator‐dependent variability [11]. The digitally designed outcome was then transferred to the clinical setting using a 3D‐printed model and a silicone index. This facilitated accurate reproduction of palatal contour, incisal edge position, and proximal boundaries during direct composite placement. The guided transfer technique has been demonstrated to reduce reliance on freehand adjustment and improve the reproducibility of the planned esthetic design in a clinical environment [12].

In this case study, a total of 7.281 mm of interdental space was redistributed across six anterior teeth (13–23). Consequently, the width‐to‐height ratios of several restored teeth exhibited deviations from the ideal esthetic proportions, including the classical golden proportion range. This represents a necessary clinical compromise to achieve complete space closure within a single minimally invasive restorative approach, and reflects the balance between esthetic ideals and functional clinical constraints. The findings indicate that attaining the theoretically ideal proportions is not always a viable objective in complex anterior esthetic reconstruction. A more pragmatic approach would entail an emphasis on attaining concordant and consistent clinical outcomes, with these outcomes being informed by and contingent upon individual circumstances [13, 14].

The present case is predicated on the precise morphological control that has been achieved through digital design. The investigation further focused on the biological impact of proximal restoration on the interdental papilla. In the event of closure of multiple interdental spaces, there is a risk of excessive proximal overcontouring or inadequate embrasure morphology, which may result in compression of the interdental papilla. This may lead to postoperative gingival inflammation and esthetic deficiencies [15]. In this case, the position and contour of the proximal contact areas were planned during the digital preoperative design phase of the restorations. At the 24‐month follow‐up, the gingival papilla index of all restored sites remained ≤ Grade 2 according to the Jemt classification [16]. The gingival papillae are well‐filled, with no dark triangles visible, consistent with the preoperative plan.

Previous studies have reported the use of digitally fabricated printed guides combined with injectable flowable composite systems for the restoration of anterior tooth defects, particularly in cases involving relatively simple morphological conditions such as single diastema closure [17]. These techniques enable a predictable transfer of planned anatomy and have demonstrated satisfactory esthetic outcomes in selected indications. In contrast, the present case employed a digitally guided silicone index in combination with direct composite resin layering. This approach facilitated the concurrent reconstruction of fractured tooth morphology and systematic closure of multiple diastemas under a unified digital spatial plan. A comparison of the layering approach with injectable flowable composite techniques reveals that the former provides superior control over anatomical detail and optical layering. However, this approach remains technique‐sensitive and requires adequate operator experience and training in both digital planning and direct composite layering [18]. Its clinical accuracy also depends on reliable digital scanning and precise transfer procedures, as deviations during scanning or index fabrication may affect the final outcome. In addition, the durability of the restorations beyond the 24‐month follow‐up period requires further evaluation.

## 6 Conclusion

This digitally guided strategy may offer a conservative treatment option for anterior esthetic rehabilitation involving crown fractures and multiple diastemas and may be considered in selected young patients seeking an alternative to prolonged orthodontic or indirect restorative treatment. Its application is dependent on digital technology and operator expertise. Further clinical studies with larger samples and longer follow‐up are needed to validate its predictability and long‐term performance.

## Author Contributions

**Chang Liu:** conceptualization, writing – original draft, writing – review and editing, investigation, methodology. **Binghua Lv:** conceptualization, writing – original draft, writing – review and editing. **Huimin Nie:** data curation, investigation, project administration. **Xiangyu Ji:** data curation, investigation, project administration. **Zhishan Lu:** project administration, supervision, writing – review and editing.

## Funding

The authors have nothing to report.

## Consent

Written informed consent was obtained from the patient to publish this report in accordance with the journal's patient consent policy.

## Conflicts of Interest

The authors declare no conflicts of interest.

## Data Availability

The data supporting this study's findings are available upon reasonable request from the corresponding author.
